# Bilateral downregulation of Nav1.8 in dorsal root ganglia of rats with bone cancer pain induced by inoculation with Walker 256 breast tumor cells

**DOI:** 10.1186/1471-2407-10-216

**Published:** 2010-05-20

**Authors:** Xue-Rong Miao, Xiao-Fei Gao, Jing-Xiang Wu, Zhi-Jie Lu, Zhang-Xiang Huang, Xiao-Qing Li, Cheng He, Wei-Feng Yu

**Affiliations:** 1 Department of Anesthesiology, Eastern Hepatobiliary Surgery Hospital, Second Military Medical University, Shanghai, China; 2 Institute of Neuroscience and Key Laboratory of Molecular Neurobiology, Ministry of Education, Second Military Medical University, Shanghai, China

## Abstract

**Background:**

Rapid and effective treatment of cancer-induced bone pain remains a clinical challenge and patients with bone metastasis are more likely to experience severe pain. The voltage-gated sodium channel Nav1.8 plays a critical role in many aspects of nociceptor function. Therefore, we characterized a rat model of cancer pain and investigated the potential role of Nav1.8.

**Methods:**

Adult female Wistar rats were used for the study. Cancer pain was induced by inoculation of Walker 256 breast carcinosarcoma cells into the tibia. After surgery, mechanical and thermal hyperalgesia and ambulation scores were evaluated to identify pain-related behavior. We used real-time RT-PCR to determine Nav1.8 mRNA expression in bilateral L4/L5 dorsal root ganglia (DRG) at 16-19 days after surgery. Western blotting and immunofluorescence were used to compare the expression and distribution of Nav1.8 in L4/L5 DRG between tumor-bearing and sham rats. Antisense oligodeoxynucleotides (ODNs) against Nav1.8 were administered intrathecally at 14-16 days after surgery to knock down Nav1.8 protein expression and changes in pain-related behavior were observed.

**Results:**

Tumor-bearing rats exhibited mechanical hyperalgesia and ambulatory-evoked pain from day 7 after inoculation of Walker 256 cells. In the advanced stage of cancer pain (days 16-19 after surgery), normalized Nav1.8 mRNA levels assessed by real-time RT-PCR were significantly lower in ipsilateral L4/L5 DRG of tumor-bearing rats compared with the sham group. Western-blot showed that the total expression of Nav1.8 protein significantly decreased bilaterally in DRG of tumor-bearing rats. Furthermore, as revealed by immunofluorescence, only the expression of Nav1.8 protein in small neurons down regulated significantly in bilateral DRG of cancer pain rats. After administration of antisense ODNs against Nav1.8, Nav1.8 protein expression decreased significantly and tumor-bearing rats showed alleviated mechanical hyperalgesia and ambulatory-evoked pain.

**Conclusions:**

These findings suggest that Nav1.8 plays a role in the development and maintenance of bone cancer pain.

## Background

Rapid and effective treatment of cancer-induced bone pain that diminishes the quality of life of affected patients remains a clinical challenge. Metastasis of tumor cells to bone is particularly common in patients with lung, breast, and prostate cancer [1]. The presence of bone metastases predicts the occurrence of pain and is the most common cause of cancer-related pain. Although bone metastases do not involve vital organs, they may have deleterious effects in patients with prolonged survival. The greatest obstacle to the development of new treatments for cancer pain is our limited knowledge of the basic neurobiological mechanisms that lead to cancer pain [2]. Recently, breast cancer, prostate cancer and sarcoma cells were used to successfully induce cancer pain in rats. These models have furthered our understanding of the mechanisms underlying cancer pain.

Voltage-gated sodium channels (VGSCs) are essential in regulating the excitability of neurons and significant changes in the expression of these channels can produce abnormal spontaneous firing patterns that can lead to chronic pain [3,4]. Sensory neurons express several VGSC subunits with fast (e.g. Nav1.3 and Nav1.7) or slow (Nav1.8 and Nav1.9) kinetics [5-8]. Whereas fast VGSCs are selectively blocked by the puffer-fish poison tetrodotoxin (TTX), both Nav1.8 and Nav1.9 are resistant to TTX (TTX-R). Nav1.8, formerly called the sensory neuron-specific or the peripheral nerve sodium channel type 3, is expressed exclusively in sensory neurons and is not found in the central nervous system (CNS) [5]. Nav1.8 channels produce the majority of the inward current during the action potential (AP) upstroke in the dorsal root ganglion (DRG) neurons in which they exist [9], most of which are nociceptors [10]. Changes in the expression, trafficking, and redistribution of Nav1.8 in chronic pain models are considered to account for abnormal firing and the generation of ectopic activity in afferent nerves [11]. Carrageenan-induced inflammatory pain increases Nav1.8 mRNA expression and the density of TTX-R currents [12]. Peripheral axotomy results in a decrease in the expression of functional Nav1.8 channels and TTX-R currents in C-type DRG neurons, suggesting a basis for the altered electrical properties observed after peripheral nerve injury [13]. Moreover, antisense oligonucleotide-mediated in vivo knockdown of Nav1.8 after intrathecal administration leads to a marked decrease in neuropathic [14] and inflammatory pain [15]. Genetic engineering approaches revealed a critical role of NaV1.8 in mediating pathologic pain: Nav1.8 knockout mice exhibited pronounced analgesia to noxious mechanical stimuli and delayed development of inflammatory hyperalgesia while exhibiting generally normal behavior [16]. However, the Nav1.8 null mice showed normal hyperalgesia in the early days after partial ligation of sciatic nerve[17]. It was recently reported that a Nav1.8-selective compound (A-803467) produces significant antinociception in animal models of neuropathic and inflammatory pain, implying that Nav1.8-targeted chronic pain therapy should be feasible [18]. These results indicate that Nav1.8 plays an important role in nociception and raise the possibility that it is involved in chronic cancer pain. However, the expression of Nav1.8 and the role it plays in cancer pain are still unclear.

In this study, we hypothesized that Nav1.8 contributes to nociceptive hypersensitivity in a cancer pain model induced by bone metastases of Walker 256 breast carcinosarcoma cells. To test this hypothesis, we examined Nav1.8 mRNA levels and protein expression in DRG of rats with tumors. Moreover, antisense oligodeoxynucleotides (ODNs) against Nav1.8 were administered intrathecally to knock down Nav1.8 expression and explore the effect on pain-related behavior.

## Methods

### Animals

All experiments were performed in accordance with the NIH Guide for the Care and Use of Laboratory Animals and the Ethical Issues of the IASP [19] and were approved by the Second Military Medical University Committee on Animal Care. Adult female Wistar rats (200-250 g) used for the study were housed under a 12-h light/dark cycle in a pathogen-free area with *ad libitum *access to water and food.

### Preparation of Walker 256 cells

Walker 256 carcinosarcoma breast cancer cells were kindly provided by the Shanghai Institute of the Pharmaceutical Industry. They were prepared and developed as previously described [20]. In brief, Walker 256 tumor cells were obtained from an ascitic tumor-bearing rat and collected by centrifugation of 2 mL of ascitic fluid for 3 min at 1200 rpm. The resulting pellet was washed twice with 10 mL of Dulbecco's modified Eagle medium (Biosource, Camarillo, CA, USA). The final pellet was resuspended in 3 mL of PBS solution and cells were counted using a hemocytometer and trypan blue solution. Cells were diluted to the final concentration for injection and kept on ice. For the sham group, Walker 256 cells were prepared at the same final concentrations for injection and boiled for 20 min.

### Induction of bone cancer

Animals subjected to tumor cell implantation were anesthetized by intraperitoneal injection of sodium pentobarbital (40 mg/kg). The left leg was shaved and the skin was disinfected with 70% (v/v) ethanol. A 1-cm-long rostro-caudal incision was made in the skin over the lower one-third of the tibia for easy exposure with minimal damage to muscles and nerves. The medullary canal was approached by inserting a 23-gauge needle proximally through a hole drilled in the tibia. The needle was then replaced with a 20-μL microinjection syringe containing the cells to be injected. A 10-μL volume of Walker 256 cells (2 × 10^5 ^cells) or boiled cells (sham group) was injected into the bone cavity. After a 2-min delay to allow cells to fill the bone cavity, the syringe was removed and the drill hole was sealed using bone wax. The wound was closed using 1-0 silk threads and dusted with penicillin powder. The rats were allowed unrestricted movement in their cages after recovery and their general condition was monitored during the experiment.

### ODN administration

Rats were anesthetized and an intrathecal catheter was inserted according to the method of Storkson et al. [21]. In brief, a PE-10 polyethylene catheter was implanted into the subarachnoid space between the L5 and L6 vertebrae to reach the lumbar enlargement of the spinal cord at a depth of 1.5-2 cm. The outer part of the catheter was plugged and fixed onto the skin on closure of the wound. The incision was sutured at all levels and 2000 U of penicillin was administered to prevent infection. Animals were observed for 3 days after catheter insertion and those without any obvious change in movement were chosen for intrathecal administration. A Nav1.8 antisense ODN (5-GGG GAG CTC CAT CTT CTC-3) (Integrated DNA Technologies, Coralville, IA) was directed against a unique sequence of the Nav1.8 sodium channel [22]. The mismatch ODN sequence, 5-GGG G**TC T**TC CA**A GC**T CTC-3, was derived from the antisense sequence by scrambling six bases (denoted in bold font). ODNs were lyophilized and reconstituted in 0.9% NaCl prepared with nuclease-free water to a concentration of 2 μg/μL. ODNs or saline were intrathecally injected via the implanted catheter in a 10-μL volume of solution followed by 15 μL of saline for flushing. Daily intrathecal administration started on day 14 after tumor inoculation for 3 days and pain behavior was assessed every 2 or 3 days.

### Mechanical hyperalgesia test

To assess mechanical hyperalgesia, animals were acclimated daily for 10 min/day for 3 days to the test environment, which was a Plexiglass box on a metal grid surface. On test days, rats were allowed to acclimate for 5-10 min. The nociceptive stimulus, a single rigid filament attached to a hand-held transducer (electronic Von Frey anesthesiometer; IITC, Woodland Hills, CA) was applied perpendicularly to the medial surface of the hind paw with increasing force. The endpoint was taken as nocifensive paw withdrawal accompanied by head turning, biting and/or licking. As soon as this reaction occurred, the required pressure was indicated in grams, and this value was considered to be the individual paw withdrawal threshold (PWT) value. Both hind limbs of each rat were tested in triplicate per time point and the average for the three measurements was then calculated.

### Hargreaves test

Paw withdrawal latency (PWL) was measured for both paws as previously described [23] before any procedure. In brief, rats were placed under an inverted clear plastic chamber on a glass surface. After an adaptation period of 30 min, a radiant heat stimulus was applied to the plantar surface of each hind paw from underneath the glass floor using a projector lamp bulb (automatic plantar analgesia tester; Institute of Biomedical Engineering, Chinese Academy of Medical Science, Tianjin, China). The radiant heat intensity was adjusted so that the PWL for normal rats was 10 ± 2 s. A cutoff time of 20 s was imposed to prevent tissue damage. Paws were alternated randomly to preclude order effects and PWL was determined as the mean of three measurements per paw.

### Ambulatory-evoked pain

Rats were placed in a large plastic observation box with a smooth floor. According to the extent of limb use during spontaneous ambulation, scores were characterized as follows: 0, normal use; 1, slight limp; 2, severe limp; or 3, complete lack of limb use. Testing was blind with respect to group.

### Immunofluorescence

Rats were anesthetized and perfused transcardially with normal saline followed by 4% paraformaldehyde in 0.1 mol/L phosphate buffer (pH 7.4) at 16-19 days after Walker 256 cell inoculation or 1 day after intrathecal administration. L4/L5 DRG were immediately dissected and postfixed in 4% paraformaldehyde in 0.1 M PBS, pH 7.2 for 2-4 h. The DRG were then transferred to 30% sucrose in PBS and kept in the solution until they sank to the bottom. Sections (7 μm) were cut using a Leica CM1900 cryostat and thaw-mounted in series on 10 slides, with each slide containing 6-8 sections at different levels throughout the DRG. Slides were washed in PBS, blocked in 10% normal horse serum (NHS) in PBS for 30 min and incubated with polyclonal rabbit anti-Nav1.8 antibody (Chemicon, CA, USA) diluted 1:200 in antibody dilution solution (10% NHS and 0.4% sodium azide in PBS) overnight. The sections were then incubated in FITC-conjugated donkey anti-rabbit IgG (Jackson ImmunoResearch Lab, West Grove, PA) at a dilution of 1:400 in antiserum diluent. Images were recorded using a DXM1200 digital camera (Nikon, Japan) attached to an Eclipse E600 microscope (Nikon). Images were imported into Adobe Photoshop CS3. The Nav1.8 immunocytochemical intensity was measured using a semiquantitative method [10]. In brief, the total area of a neuron or cytoplasm was manually selected and the neuronal cross-sectional area (including the nucleus) and mean pixel density for immunostaining in the cytoplasm (excluding the nucleus) were determined by the software. For each image, the relative intensity for each neuron was calculated using the normalized method previously described [24] and a relative intensity of 20% was used as the threshold to discriminate between negative and positive neurons. As defined previously [25], neurons with a cross-sectional area of up to 400 μm^2 ^were classified as small (diameter 23 μm), those of >800 μm^2 ^(diameter 32 μm) as large, and those of 400-800 μm^2 ^(diameter 23-32 μm) as medium-sized.

### Western blotting

Rats were deeply anesthetized with 40 mg/mL sodium pentobarbital and killed by decapitation at 16-19 days post surgery. L4/L5 DRG were removed, homogenized and lysed in a solution of 2% SDS, 20% glycerol, and 100 mM Tris-HCl, pH 6.8 containing Complete protease inhibitor (Roche Diagnostics, Rotkreuz, Switzerland). The homogenate was centrifuged for 10 min at 10,000 rpm at 4°C, the supernatant was recovered and the protein concentration was determined by the Bradford method. The sample was diluted in sample buffer (250 mm Tris-HCl, pH 6.8 containing 4% SDS, 10% glycerol, 2% β-mercaptoethanol, and 0.002% bromophenol blue) and boiled for 10 min. A sample aliquot was separated by SDS-PAGE (10%) and then transferred to a nitrocellulose membrane by electroblotting. The membrane was blocked in 5% skim milk powder in 0.1% Tris-buffered saline/Tween 20 at room temperature for 2 h, and then incubated with antibody raised against Nav1.8 (Chemicon Int., CA, USA) at a dilution of 1:500 overnight at 4°C. Antibody binding was visualized using a horseradish peroxidase-conjugated secondary antibody and an enhanced chemiluminescence Western blotting detection system (Santa Cruz Biotechnology, Santa Cruz, CA). Light-emitting bands were detected using X-ray film. The band intensities were quantitated using an image scanning densitometer (Furi Technology, Shanghai, China). To control sampling errors, the Nav1.8/β-actin band intensity ratio was obtained to quantify relative protein expression levels.

### Total RNA extraction and quantitative real-time RT-PCR

Tumor-bearing and sham rats were deeply anaesthetized and killed by decapitation at 14-18 days post surgery. L4/L5 DRG were collected and mechanically dispersed by scraping with a rubber policeman for 1 min in the presence of TRIzol reagent (Invitrogen, Grand Island, NY) and then incubated for 5 min at the room temperature for complete nucleoprotein dissociation. Total RNA was prepared from individual samples using TRIzol reagent according to the manufacturer's instructions. The purity and integrity of the RNA were checked spectroscopically and by gel electrophoresis before use. A sample of 2 μg of RNA was reverse transcribed with oligo (dT)18 primer using Moloney murine leukemia virus reverse transcriptase (Promega, Madison, WI, USA). The primer sequences for Nav1.8 were: sense, 5'-GGA CTC CCT GAA GAC CAA TAT GGA AG-3'; antisense 5'-GCA TTG AGC TAG ATG GGT TAA TGT TG-3'. This should amplify a fragment of 361 bp corresponding to nucleotides 5479-5840 of the Nav1.8 coding region (GenBank accession number U53833). Quantitative real-time PCR analysis was carried out using a Rotor Gene 3000 system (Corbett Research, Sydney, Australia). The reaction solution consisted of 2.0 μL of diluted cDNA product, 0.2 μM of each paired primer, 200 μM deoxynucleotide triphosphates, 1 U of Taq DNA polymerase (Promega), and 1× PCR buffer. SYBR Green dye (BMA, Rockland, ME, USA) was used for detection. PCR conditions were optimized in a preliminary experiment to achieve a linear relationship between the initial RNA concentration and the PCR product. The annealing temperature was 55-58°C and 40 amplification cycles were carried out. The range for detection of the melting temperature of the PCR product was 60-95°C. Amplification of the housekeeping gene β-actin was measured for each sample as an internal PCR control for sample loading and normalization. The specificity of the primers was verified by examining the melting curve and subsequent sequencing of the real-time PCR products. To quantitate the relative amount of gene expression for the target and housekeeping genes, the comparative threshold cycle (Ct) method was used. Subtracting Ct for the housekeeping gene from Ct for the target gene yields ΔCt in each group (control and experimental groups), which was entered into the equation 2^-ΔCt ^and calculated for exponential PCR amplification. Nav1.8 mRNA levels were normalized relative to β-actin values to ensure that a linear relationship between the initial RNA concentration and the PCR product was achieved in each run.

### Data analysis

Data from most assays were analyzed using one-way measures analysis of variance (ANOVA) followed by post-hoc Scheffe's multiple comparisons. Immunofluorescence data were analyzed by **χ**^2 ^test. P < 0.05 was set as the level of statistical significance in all cases.

## Results

### General observations

All animals displayed general good health with no signs of distress during the 21-day observation period. Signs of tumor growth, observed as swelling around the tibia, became visible in most cancer animals by day 14. As shown in a related study in this issue [20], Walker 256 carcinoma causes progressive destruction of the calcaneus bone by this time.

### Tumor inoculation-induced pain model

**Figure 1:**
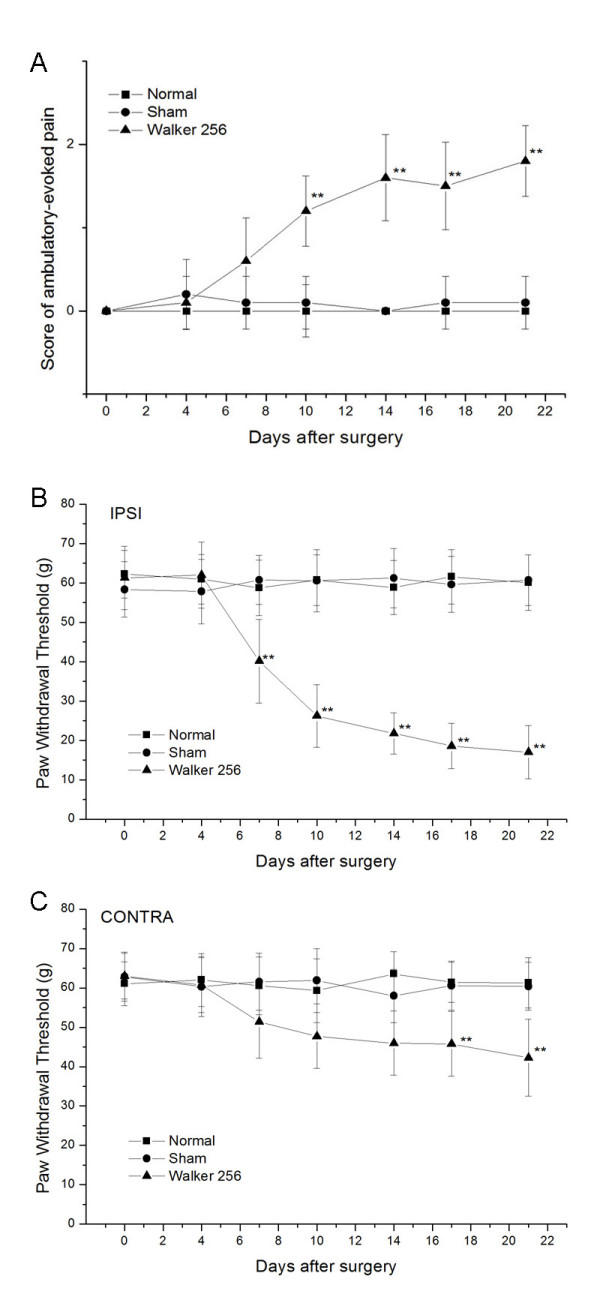
**Time course of pain-related behavior in rats of the Walker 256 group (n = 10), the sham group (n = 10) and the normal group (n = 8)**. (A) The ambulatory score increased on days 10, 14, 17 and 21 after intra-tibial injection of Walker 256 carcinoma cells but not after injection of heat-killed cells. Data are expressed as mean ± SD. **P < 0.01 compared to normal rats. (B) After Walker 256 carcinoma cell inoculation, the PWT for the inoculated hind paw progressively decreased as revealed by electronic Von Frey filament stimulus. Data are mean ± SD. **P < 0.01 compared to normal rats. (C) The PWT for the contralateral hind paw of tumor-bearing rats changed slightly but significantly on days 17 and 21 Data are expressed as mean ± SD. **P < 0.01 (ANOVA) compared to normal rats.

There was no evidence of ambulatory pain in animals that received a sham injection. By contrast, animals injected with Walker 256 carcinoma cells exhibited an apparent limp on the injected hind limb over days following injection. A significant (P < 0.01) decrease in ipsilateral limb use by tumor-bearing rats was observed from day 10 after surgery (Fig. 1A).

Tumor-bearing rats showed pronounced curling of the toes, cupping and guarding of the ipsilateral paw, and a distinct preference for weight bearing on the contralateral hind paw during normal ambulation on a metal grid surface as part of the mechanical hyperalgesia test. The baseline PWT was 59.8 ± 7.5 and 62.9 ± 5.8 g for the left and right hind paw, respectively. After Walker 256 cell inoculation, the PWT of both paws decreased progressively (Fig. 1B, C). Post-hoc mean comparisons revealed that cancer cell inoculation of the tibia induced a significant (P < 0.01) decrease in PWT for the ipsilateral hind paw from day 7 after surgery. The PWT for the contralateral hind paw of tumor-bearing rats changed slightly but significantly from day 17 (P < 0.05) compared to the normal group, suggesting the development of mirror-image pain. Rats injected with heat-killed cells showed no changes in PWT for both hind paws.

No significant (P > 0.05) thermal hyperalgesia was observed in tumor-bearing rats on either side compared with sham or normal rats. These findings are in agreement with previous reports [20,26].

### Downregulation of Nav1.8 in cancer pain rats

**Figure 2:**
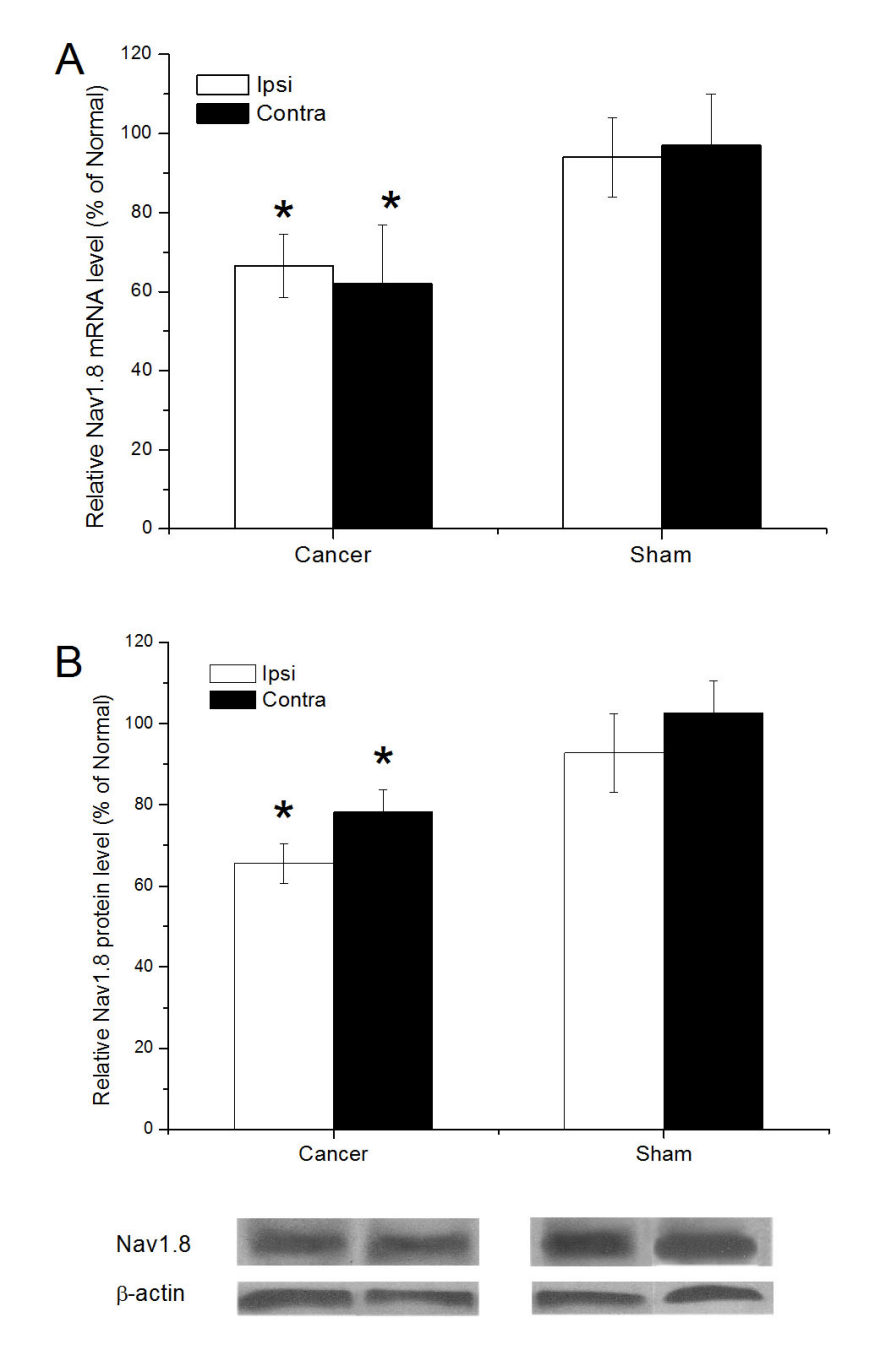
**Regulation of Nav1.8 mRNA and protein levels in bilateral DRG of tumor-bearing and sham rats**. (A) Relative quantification (triplicate analysis of 4-6 rats for each time point) by real-time RT-PCR revealed a significant decrease in Nav1.8 mRNA in bilateral DRG of tumor-bearing rats compared with the sham group. (B) Western blots revealed a significant decrease in bilateral DRG of tumor-bearing rats compared with the sham group. Moreover, Nav1.8 protein expression was significantly downregulated on the ipsilateral side compared with the contralateral side of tumor-bearing rats. Data are expressed as mean ± SD. *P < 0.05 (one-way ANOVA) compared with sham rats.

We used real-time RT-PCR to compare Nav1.8 mRNA expression in L4/L5 DRG 16-19 days after injection (Fig. 2A). Nav1.8 transcript levels were normalized to β-actin and expressed as the ratio of the Nav1.8 level in DRG from tumor-bearing or sham rats to the level in DRG from normal rats. Injection of Walker 256 cells resulted in significant downregulation of Nav1.8 mRNA in bilateral DRG of tumor-bearing rats compared with the sham group (P < 0.05). In sham animals, no significant changes were observed in Nav1.8 mRNA expression compared to normal rats (normal data not shown).

Western blotting (Fig. 2B) revealed a significant decrease in relative Nav1.8 protein levels (percentage of normal) in bilateral DRG of tumor-bearing rats (65.6 ± 4.9 and 78.2 ± 5.4 for ipsilateral and contralateral respectively) compared to the sham group (P < 0.01). Moreover, a post hoc test revealed that Nav1.8 protein expression on the ipsilateral side of tumor-bearing rats was significantly downregulated (P < 0.05) compared with the contralateral side.

**Figure 3:**
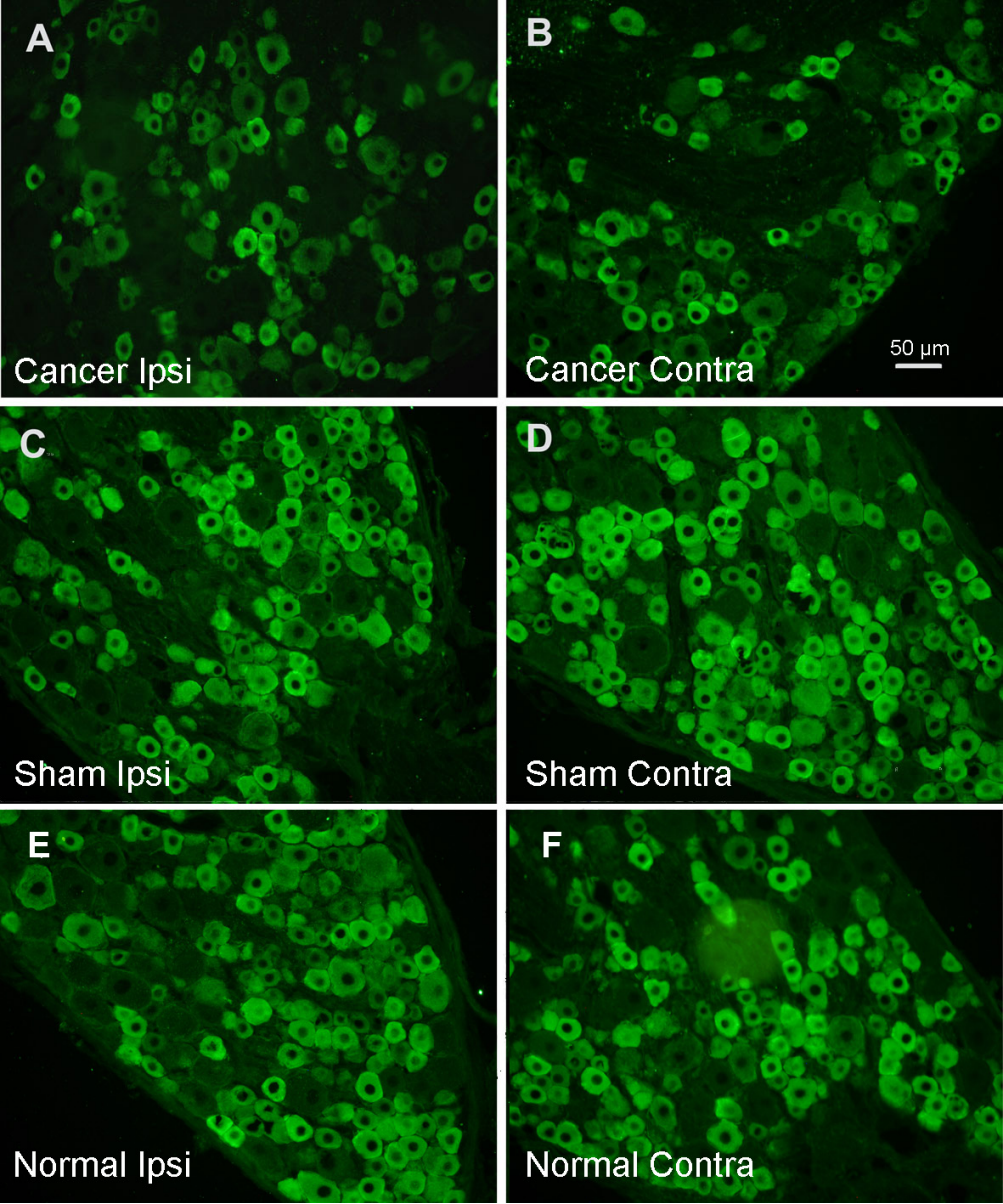
**Representative images of Nav1.8 immunofluorescence in bilateral DRG from rats of the Walker 256 group, the sham group and the normal group**. (A) Ipsilateral and (B) contralateral DRG from a tumor-bearing rat. (C) Ipsilateral and (D) contralateral DRG from a sham rat. (E) Ipsilateral and (F) contralateral DRG from a normal rat. Note that Nav1.8 expression in tumor-bearing rats decreased compared to the normal group, although it is not clear from a simple vision inspection whether expression was lower on the ipsilateral or the contralateral side, highlighting the need for quantitative analysis. Scale bar, 50 μm.

**Figure 4:**
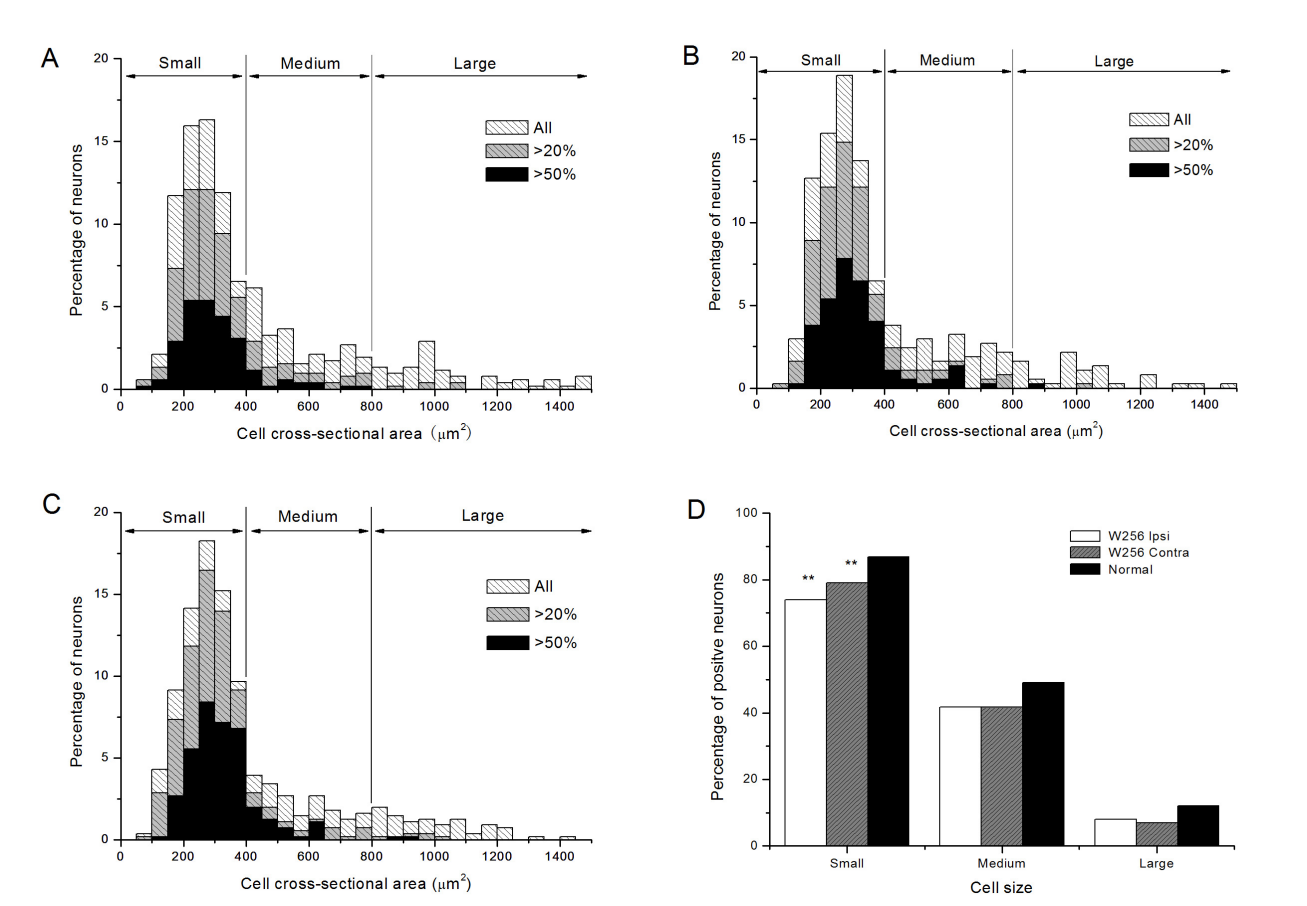
**Cell area and relative Nav1.8 immunofluorescence intensity**. Histograms for neurons in tumor-injected (A) ipsilateral (n = 521), (B) contralateral (n = 449) and (C) normal DRG (n = 549). Grey and black denote neurons with Nav1.8 immunofluorescence relative intensity >20% and >50%, respectively. Neurons are divided into small (cross-sectional area <400 μm^2^, diameter 23 μm), medium (400-800 μm^2^, diameter 23-32 μm) and large (>800 μm^2^, diameter 32 μm). (D) Semiquantitative analysis of the percentage of Nav1.8 immunofluorescent-positive (>20% relative intensity) cells in small, medium and large DRG neurons. The percentage of positive small neurons is significantly (P < 0.01) lower in tumor-injected bilateral DRG compared to the normal group. No differences are evident for medium and large neurons. *P < 0.01 (**χ**^2 ^test) compared to normal rats.

In addition, we investigated changes in Nav1.8 distribution in DRG by immunofluorescence 16-19 days after injection. Nav1.8 expression was lower in tumor-bearing rats than in the sham group (Fig. 3). Since no differences were observed between sham and normal rats, we choose normal rats as the control group in the next semiquantitative analysis. Histograms for neurons (Fig. 4) showed the cell area and Nav1.8 immunofluorescence relative intensity. semiquantitative analysis showed the percentage of Nav1.8 immunofluorescent positive (>20% maximum intensity) cells in small, medium and large DRG neurons. There was a significant decrease (P < 0.01) in the percentage of Nav1.8-positive small neurons in bilateral DRG from the tumor-bearing group accompanied by advanced cancer pain compared to the sham group; the decrease was from 86.9% to 74.0% for the ipsilateral side and to 79.0% for the contralateral side. However no effects were observed in the percentage of Nav1.8-positive cells in medium and large neurons.

### Knockdown of Nav1.8 attenuates cancer pain behavior

The expression of Nav1.8 in DRGs decreased in cancer pain rats, which is similar to those in neuropathic models[11,13,14]. Therefore we decided to knockdown Nav1.8 referring to the study approaches of Nav1.8 in neuropathic pain models[14].

**Figure 5:**
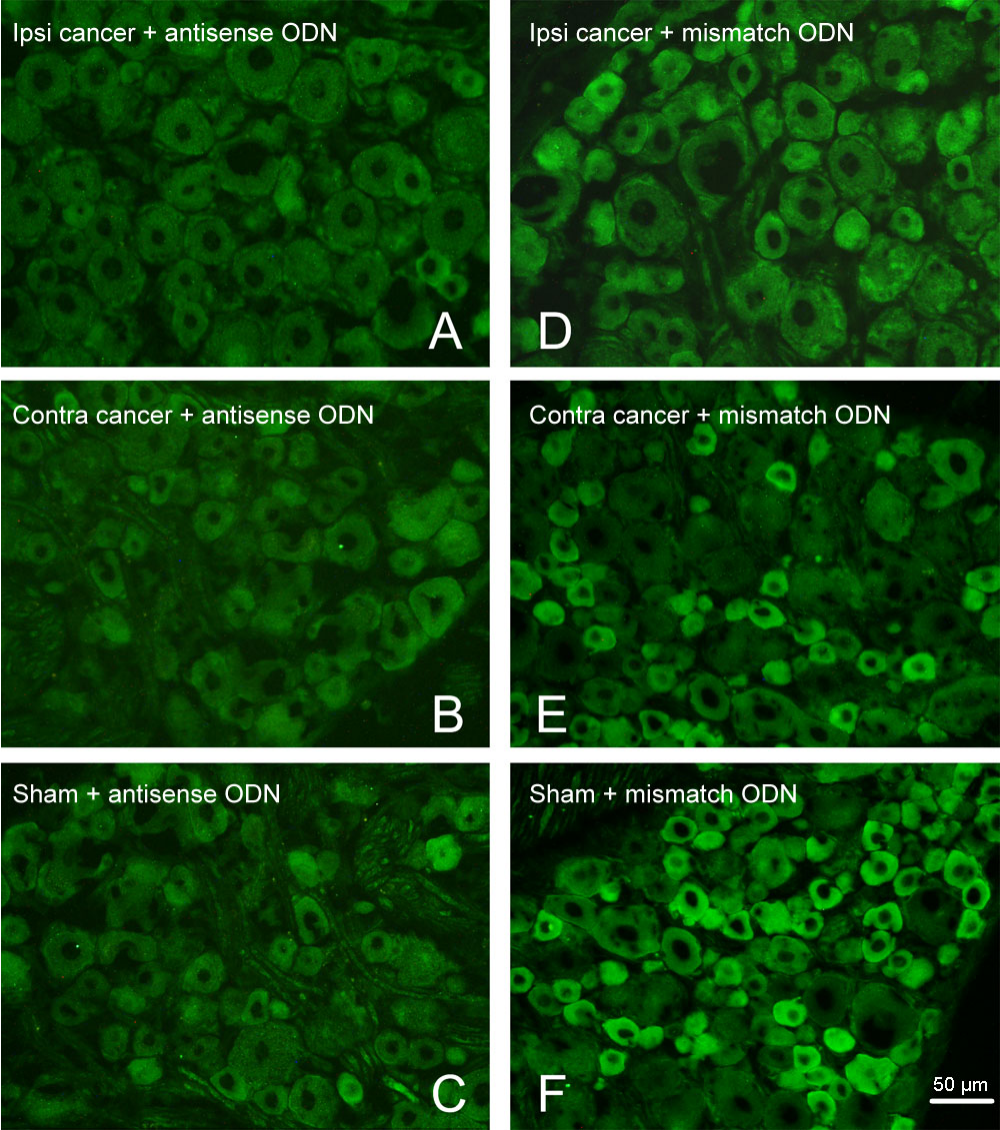
**Representative photomicrographs of Nav1.8 immunofluorescence in DRG from cancer and sham rats after ODN injection**. DRG contained significantly fewer Nav1.8-positive neurons in animals that received antisense ODNs (A-C) compared with animals that received mismatch ODNs (D-F). Scale bar, 50 μm.

To verify the effect of antisense ODNs, Nav1.8 expression in L4/L5 DRG was measured following intrathecal delivery of ODNs or saline once daily for 3 days. Immunofluorescence analysis revealed that antisense, but not mismatch, ODNs significantly decreased Nav1.8 immunoreactivity in DRG compared with saline control rats (Fig. 5). The resulting data confirm that Nav1.8 expression in DRG can be significantly downregulated by antisense ODN treatment.

**Figure 6:**
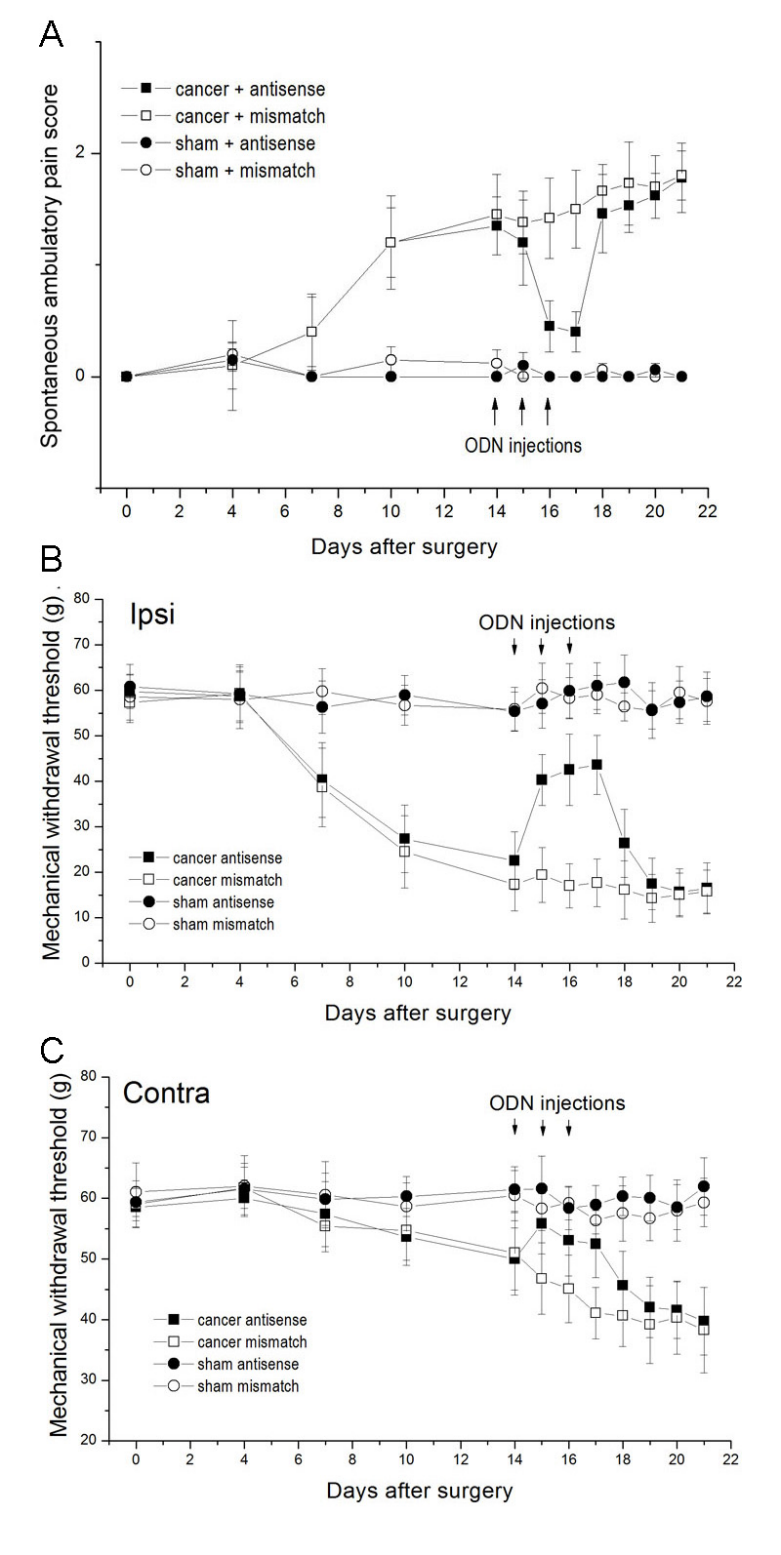
**Time course of pain-related behavior in rats after intrathecal injection of antisense or mismatch ODNs**. (A) Antisense, but not mismatch, ODNs to Nav1.8 administered by intrathecal injection alleviated ambulatory-evoked pain in cancer rats. There were no changes in ambulatory-evoked pain scores in sham rats that received antisense ODNs. Ambulatory-evoked pain scores were reversed by approximately 48 h after antisense ODN administration and returned on the second day after the last Nav1.8 antisense ODN injection (n = 7 each group). Consistently, antisense, but not mismatch, ODNs to Nav1.8 administered by intrathecal injection alleviated established mechanical hyperalgesia in ipsilateral (B) and contralateral (C) paws of in cancer rats. Mechanical hyperalgesia was reversed by approximately 48 h after the antisense ODN administration. The hyperalgesia returned on the second day after the last Nav1.8 antisense ODN injection.

The functional consequences of Nav1.8 knockdown by antisense ODNs were evaluated in pain-related behavioral tests (Fig. 6). The absence of any abnormal behavioral characteristics during ODN treatment demonstrates that the ODN dosage and the various sequences used did not precipitate any non-specific, sequence-independent, ODN-mediated behavioral toxicity. Nav1.8 knockdown had no effect on the CNS and other organ systems, as Nav1.8 is expressed exclusively in primary afferent neurons.

Neither antisense nor mismatch ODNs to Nav1.8 had any effect on ambulatory pain in sham-operated rats. In addition, neither antisense nor mismatch ODNs altered the response of sham-operated rats to nociceptive mechanical or thermal stimuli, as revealed by mechanical hyperalgesia and Hargreaves tests.

In rats with cancer pain induced by breast cancer cell inoculation, treatment with mismatch ODNs had no significant effect on pain-related behavior. By contrast, antisense ODN administration reversed ambulatory pain scores (P < 0.05) and PWT (P < 0.05) on the ipsilateral side from approximately 2 days after administration. The analgesia effect disappeared on the second day after the last antisense ODN administration. PWT on the contralateral side increased after antisense ODN administration, but only reached significance on the first day after the last administration. No changes in thermal PWL were observed after antisense ODN administration in rats with cancer pain.

## Discussion

This study shows that further reduction of Nav1.8 reduced hypersensitivity and pain. Nav1.8 mRNA and protein levels decreased in ipsilateral DRG neurons, especially in small neurons, concomitantly with the occurrence of stable ambulatory-evoked pain and mechanical hyperalgesia. Moreover, antisense, but not mismatch, ODNs to Nav1.8 administered by intrathecal injection alleviated established pain-related behavior in tumor-bearing rats. This decrease in nociceptive sensitivity was accompanied by a significant decrease in Nav1.8 in DRG neurons after injection of antisense ODNs to Nav1.8.

These results lend further credence to earlier conclusions that Nav1.8 plays a critical role in many aspects of nociceptor function [14,27-30]. Different Nav1.8 expression patterns have been observed in different animal models of chronic peripheral pain, including bone cancer pain induced by inoculation of breast cancer cells in rats. In addition, downregulation of Nav1.8 in ipsilateral DRG neurons in rats with cancer pain is similar to that in rats with peripheral neuropathic pain, suggesting the importance of a neuropathic element in bone cancer pain. Tumor-induced injury of sensory nerve fibers has been observed in ipsilateral DRG of tumor-bearing mice [31], suggesting that afferent nerve injury in a manner mimicking neuropathic pain is a probable cause of the decreased ipsilateral expression of Nav1.8.

In this cancer pain model of rats, the contribution of the Nav1.8 channel activity to the maintenance of cancer pain does not appear to be directly related to an enhanced synthesis of Nav1.8 in the primary afferent nerve. The physiological properties of the TTX-resistant persistent current associated with Nav1.8, which include a broad overlap between activation and steady-state inactivation centered close to resting potential, suggest that Nav1.8 contribute a depolarizing influence to resting potential[13], which in some cases, lead to a spontaneous manner of fire in the absence of stimulation which probably contribute to the maintenance of cancer pain. In addtion, Nav1.8 activity is likely to be up-regulated through other post-translational regulatory mechanisms such as the distribution of the channel proteins or protein phosphorylation[14]. It was also noted that after cancer-induced injury to sensory neurons, areas of the spinal cord and CNS involved in the processing of somatosensory information undergo various neurochemical and cellular changes, known as central sensitization, that facilitate the transmission and conscious awareness of both noxious and non-noxious sensory information [32]. Nav1.8 downregulation might also be a negative feedback effect of the CNS in a persistent severe chronic pain state.

However, we also observed downregulation of Nav1.8 in contralateral DRG neurons in tumor rats. Since no inoculation was applied to the contralateral limb, the tumorigenic component of bone cancer pain might contribute to this alteration. Intracellular Nav1.8 domains interact widely with many proteins and immunoprecipitation assays have confirmed that some of these proteins may regulate Nav1.8 *in vivo *[32]. Algogens released from a tumor or its surrounding tissue and transported all over the body, such as endothelin-1 [33], prostaglandins [34] and ATP [35], may directly or indirectly sensitize and/or excite primary afferent nociceptors and therefore interact with the proteins that regulate Nav1.8. In addition, metabolic abnormality induced by tumor development can lead to changes in sodium channel expression.

Several studies have attempted to model bone cancer pain by injecting tumor cells into the marrow space of the rodent femur or tibia. According to the model induced by Walker 256 breast carcinosarcoma cells described previously [20], we established a modified model by injecting cells into the lower tibia instead of the intercondylar eminence to avoid joint injury and for surgical precision. Tumor-bearing rats showed progressive mechanical allodynia and ambulatory-evoked pain indicative of clinical bone cancer pain.

Different tumor cells injected into bone lead to a distinct pattern of cancer-related pain behavior, skeletal destruction and neurochemical changes in the spinal cord [36]. In the current study, mechanical allodynia was observed after injection of Walker 256 cells not only in the ipsilateral hind paw, but also in the contralateral paw from day 17. These findings are in agreement with previous observations that mirror-image pain occurs in this model [20]. By contrast, no signs of mirror-image pain were observed in another model of cancer pain induced by injection of Walker 256 cells into the plantar surface of rat hind paw [26]. The reason for these differences may be that the observation period for the latter was too short (from baseline to 5-8 days after surgery) since mirror-image pain usually appears when tumors are at an advanced stage.

## Conclusions

Downregulated Nav1.8 expression was observed in DRG neurons in a cancer pain model induced by inoculation with Walker 256 breast carcinosarcoma cells. Moreover, knockdown of Nav1.8 expression attenuated cancer pain behavior. These findings suggest that Nav1.8 can contribute to the development and maintenance of bone cancer pain. Nav1.8 could be a potential target for treatment of bone cancer pain induced by metastatic breast cancer cells.

## Competing interests

The authors declare that they have no competing interests.

## Authors' contributions

W-FY and CH conceived the study, and participated in its design and coordination. Z-XH and X-QL performed animal model establishment. X-RM and X-FG performed behavior test, immunofluorescence and Western-blot. J-XW and Z-JL performed real-time RT-PCR. X-RM and X-FG conducted the statistical analysis and drafted the manuscript. All authors read and approved the final manuscript.

## Pre-publication history

The pre-publication history for this paper can be accessed here:

http://www.biomedcentral.com/1471-2407/10/216/prepub
